# Evidence of Marine Microplastics in Commercially Harvested Seafood

**DOI:** 10.3389/fbioe.2020.562760

**Published:** 2020-12-04

**Authors:** Emily Curren, Chui Pin Leaw, Po Teen Lim, Sandric Chee Yew Leong

**Affiliations:** ^1^St. John’s Island National Marine Laboratory, Tropical Marine Science Institute (TMSI), National University of Singapore, Singapore, Singapore; ^2^Bachok Marine Research Station, Institute of Ocean and Earth Sciences, University of Malaya, Bachok, Malaysia

**Keywords:** seafood, microplastic, commercial, marine, pollution, shrimp

## Abstract

Microplastic pollution is a global issue that has a detrimental impact on food safety. In marine environments, microplastics are a threat to marine organisms, as they are often the same size range as prey and are mistaken as food. Consumption of microplastics has led to the damage of digestive organs and a reduction in growth and reproductive output. In this study, microplastic pollution was assessed across three commercially available shrimp species that were obtained from the supermarkets of Singapore. A total of 93 individuals were studied from the Pacific white leg shrimp, *Litopenaeus vannamei*, the Argentine red shrimp *Pleoticus muelleri* and the Indian white shrimp *Fenneropenaeus indicus*. Microplastic fibers, fragments, film and spheres were identified from the digestive tract of these organisms. Microplastic abundance ranged from 13.4 to 7050 items. *F. indicus* exhibited the highest number of microplastics. Microplastic film was the most abundant in *L. vannamei* individuals (93–97%) and spheres were the most abundant in *P. muelleri* (70%) and *F. indicus* (61%) individuals. This study demonstrates that microplastic contamination is evident in commonly consumed shrimp and highlights the role of shrimp in the trophic transfer and accumulation of microplastics in seafood. The consumption of microplastic-containing seafood is a route of exposure to humans and has implications on human health and food security. Capsule: Microplastics were examined in three shrimp species from the supermarkets of Singapore. Microplastics ranged from 13.4 to 7050 items of shrimp.

## Introduction

Plastic pollution is a global problem of the 21st century. In marine environments, microplastics are widespread and are found in many coastal waters and even in large water bodies such as the Pacific (Desforges et al., 2014), Atlantic (Enders et al., 2015), and the Arctic (Lusher et al., 2015). Microplastics can exist as primary microplastics, which are round plastic nurdles or pellets, which are used for pre-production of larger plastic particles. Primary microplastics also exist in the form of microbeads, which were once highly popular in cosmetics such as facial cleansers and toothpastes (Napper et al., 2015). Microfibers from the effluent of washing machines also constitute primary microplastics (Kalčíková et al., 2017). Due to the small size of these particles, microplastics will evade sewage treatment and be discharged directly into the marine environment through wastewater (Pirc et al., 2016). Secondary microplastics are formed from degradation and fragmentation of larger plastic particles such as foam buoys and fishing gear due to physical, biological and chemical processes (Andrady, 2011).

Due to their small size, these plastic particles are a threat to marine life as they are often mistaken as food (Neves et al., 2015). The ingestion of microplastics has been recorded across many marine organisms, from worms (Wright et al., 2013), larvae of oysters (Cole and Galloway, 2015) and fish (Mazurais et al., 2015; Steer et al., 2017), mussels (Van Cauwenberghe et al., 2015), crabs (Watts et al., 2014), sharks (Alomar and Deudero, 2017) and whales (Lusher et al., 2015). Microplastic ingestion is detrimental, as it has led to the physical injury in organisms (Gall and Thompson, 2015), internal obstruction (Guzzetti et al., 2018), affecting their feeding behavior, reproduction and growth, In addition, the surfaces of microplastics contains heavy metals such as copper and zinc (Brennecke et al., 2016), which are harmful and can bioaccumulate in the tissues of marine animals (Jakimska et al., 2011). Leachates from these plastics contain many additives and are known to be acutely toxic to copepods (Bejgarn et al., 2015), algae (Luo et al., 2019) and gastropods (Seuront, 2018). These microplastics in the marine environment are causing harm to many wildlife organisms including marine mammals and could even result in mortalities.

The prevalence of microplastics in marine environments directly impacts commercial fisheries and aquaculture industries (Lusher et al., 2017a). Smaller commercial seafood such as shrimps or decapod crustaceans are more likely to be impacted by microplastics as compared to larger fish, because microplastics fall in a similar size range to the prey or foods of shrimp. Shrimps are filter-feeders and feed on food matter using their pereiopod (Nimrat et al., 2011) and will consume everything in their path. As a result, shrimp end up ingesting these particles by mistake and accumulate microplastics in their intestines. The accumulated microplastics will affect the safety of seafoods.

In the marine aquaculture industry, shrimp farming is commercially viable and has been adopted by over 60 countries around the world (Jory and Cabrera, 2012), including Brazil (Rebouças et al., 2011), Thailand (Nimrat and Vuthiphandchai, 2011) and Vietnam (Hai et al., 2015). In marine environments, shrimps prey on smaller organisms such as copepods (Matias-Peralta et al., 2012) and fish larvae (Theilacker and Lasker, 1974), which are also known to take in microplastics (Desforges et al., 2015; Steer et al., 2017). On the other hand, shrimps are the prey for larger marine animals such as fish (Mace and Rozas, 2018) and whale sharks (Rohner et al., 2015). Shrimps are also common and popular seafood, where many people consume them whole without gut removal. Hence, human consumption of shrimps contaminated with microplastics form a direct route of exposure, posing a threat to food security and human health. This highlights the potential role of shrimp in the trophic transfer and accumulation of microplastics in seafood.

Singapore is an island nation that produces only a small percentage of its fresh food and is largely reliant on imports, especially for seafood (Tey et al., 2009; Tortajada and Zhang, 2016). Shrimp in Singapore supermarkets originate from neighboring regions such as Malaysia, Indonesia and Vietnam, as well as from other countries such as Argentina and Australia. These shrimps are popular as seafood and commonly available in local supermarkets. For wild shrimps, there have been a few reports regarding microplastic contamination (Devriese et al., 2015; Daniel et al., 2020; Hossain et al., 2020). However, there have not been any studies investigating the presence of microplastics in commercially available shrimp in Singapore. Hence, this study aimed to elucidate the abundance and characteristics of microplastic contamination in different species of shrimp consumed in Singapore. In this study, three species of commercially farmed shrimp; the Pacific white leg shrimp, *Litopenaeus vannamei*, the Argentine red shrimp *Pleoticus muelleri* and the Indian white shrimp *Fenneropenaeus indicus* were examined for the contamination by microplastics.

## Materials and Methods

### Sample Collection

Three species of shrimp with origins from four locations were purchased from the supermarkets of Singapore in January 2020. *L. vannamei, P. muelleri*, and *F. indicus* were the shrimp species studied. A total of 93 individuals were sampled, with 30 individuals of *L. vannamei*, 15 individuals of *P. muelleri* and 18 individuals of *F. indicus*. These shrimps were imported from various countries such as Malaysia, Ecuador, Southwest Atlantic and the Indian Ocean (Table 1). These shrimp were obtained in frozen form or were thawed before purchase. Whole shrimp were used in this study, and were not deshelled or cooked before processing. Prior to experimentation, the shrimps were thawed in room temperature for 1 h, each shrimp washed with 200 ml of Milli-Q water (Merck, Millipore) and placed on clean stainless steel trays using metal forceps. The wet weight of each individual was measured using aluminum weighing boats and averaged (Table 1). Shrimps were deshelled and dissected on metal trays using metal forceps and a metal scissors. The metal forceps and scissors were washed with Milli-Q water after processing each batch of shrimps. The gastrointestinal (GI) tract was removed using metal forceps and transferred to a 100 ml glass jar. Three replicates were conducted for each species of shrimp (Table 1).

**TABLE 1:** Abundance of microplastics in different groups of shrimp.

**Species**	**Location**	**Number of individuals studied**	**Average wet weight (g w.w.)**	**Average number of microplastics/g w.w.**
*Litopenaeus vannamei*	Malaysia	30	23 ± 1	21 ± 4
	Ecuador	30	29 ± 2	13 ± 1
*Pleoticus muelleri*	Argentina Southwest Atlantic, FAO 41	15	56 ± 4	7050 ± 418
*Fenneropenaeus indicus*	Indian ocean, FAO 57	18	38 ± 1	5570 ± 100

### Contamination Control

During the experiment, shrimps were dissected and processed in a clean-air cabinet to reduce contamination. Cotton clothing, lab coats, nitrile gloves were worn during the study. Work surfaces and tools such as metal trays, metal spoons and forceps were cleaned with 70% ethanol and then rinsed with Milli-Q water before use. As far as possible, glass and metal wares were used to reduce introduction of plastic from the surrounding. For each shrimp sample studied, a black extraction control was performed without shrimp tissue to correct for any procedural contamination. This control was placed inside the clean-air cabinet while experimental procedures were carried out to account for microplastic contamination from surrounding air. Sodium hypochlorite and sodium chloride solutions were filtered using a sterile 0.22 μm syringe filter (Merck, Millipore) before use.

### Treatment of Soft Tissue With Sodium Hypochlorite

The extraction of microplastics from the GI tracts of shrimps was performed using 6.25% of filtered sodium hypochlorite (NaClO; Sujathan et al., 2017). 50 ml of NaClO was added to each glass bottle to break down the soft tissue. NaClO was chosen as the digesting solution as it is effective in digesting organic matter (Karami et al., 2017). Furthermore, other solutions such as nitric acid and hydrochloric acid are able to degrade polymers and hence are not suitable for this purpose. The glass bottles were covered with sheets of aluminum foil and placed on a flat surface at 25°C for 48 h for digestion to take place (Lusher et al., 2017b).

### Density Separation Using NaCl Solution

80 ml of saturated salt solution of pre-filtered 1.2 g/ml of sodium chloride was added to the glass jar containing digested shrimp matter for density separation of microplastics (Hidalgo-Ruz et al., 2012; Li et al., 2015). A metal spatula was used to mix the solution and left for 24 h. This enables the separation of less dense microplastics from denser organic matter such as sand and metal pieces. The top layer of solution was then gently removed and transferred into clean glass petri dishes for microscopic observation. All experimentation procedures were completed within 4 weeks of shrimp processing.

### Identification and Analysis of Microplastics

Microplastics were observed under an inverted Nikon microscope. Microplastics were assessed visually and categorized by different morphotypes such as fiber, sphere, film and fragments according to their physical characteristics. The number of microplastics were quantified and recorded.

### Statistical Analyses

Differences in microplastic abundance in shrimp types were tested using one-way analysis of variance (ANOVA) followed by Tukey’s HSD *post hoc* pairwise comparisons. A significance level of 0.01 was chosen. Statistical analyses were run using the ‘multcomp’ package (Hothorn et al., 2016) in R studio (version 1.3.1073). Reported values were corrected to three significant figures when necessary.

## Results

In this study, three species of marine shrimp, *L. vannamei, P. muelleri* and *F. indicus*, from four locations, Malaysia, Ecuador, Southwest Atlantic and the Indian Ocean were obtained from the supermarkets of Singapore and studied for their presence of microplastics. A total of 93 shrimps were sampled (Table 1). Microplastic fibers, fragments, film and spheres were detected in *L. vannamei, P. muelleri* and *F. indicus.* Pink and blue colored fibers and fragments were commonly observed. Procedural blanks revealed an average of 6 microplastics per sample, and were mostly uncolored translucent fibers.

**FIGURE 1:**
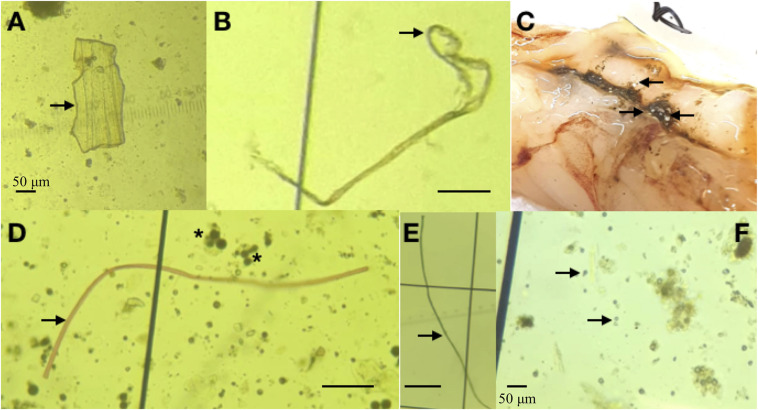
Composition of microplastics in *L*. *vannamei* shrimp (Malaysia, Ecuador), *P. muelleri* (Atlantic Ocean) and *F. indicus* (Indian Ocean). Compositions are in percentages.

The average wet weight of one individual of *L. vannamei* from Malaysia was 23 ± 1 g w.w (Table 1). A total of 14186 microplastic particles were observed in 30 individuals, with an average of 21 ± 4/g w.w. From this sample, film particles were the most abundant (97.9%; Figure 1A), followed by fragments (0.8%). Fibers and spheres were of similar abundances at 0.6%.

**FIGURE 2:**
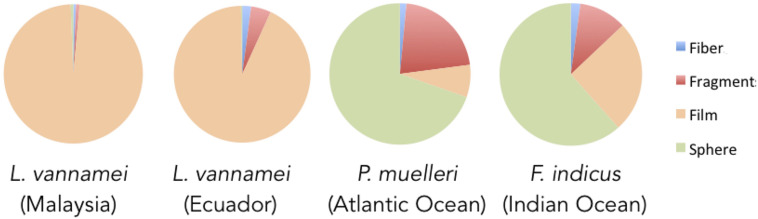
**(A-F)** Microplastic particles obtained from the digestive tracts of shrimp. **(A)** Microplastic film from *L. vannamei* (Malaysia). **(B)** Microplastic fiber from *L. vannamei* (Ecuador). **(C)** White microplastic spheres from *P. rnuelleri.* **(D)** Red microplastic fiber from *P. rnuelleri* (arrow). Agglomerations of microplastic spheres are indicated by the asterisk (*). **(E)** Blue microplastic fiber from *F. indicus.* Unless otherwise indicated, scale bars represent 20 μm.

Individuals of *L. vannamei* from Ecuador were also studied, with the average weight of one individual being 29 ± 2 g w.w. (Table 1). A total of 11625 pieces of microplastics were observed among 30 individuals, with an average of 13 ± 1/g w.w. (Table 1). From this sample, film microplastics were the most abundant (93%), followed by fragments (4.7%), fibers (2%) and spheres (0.3%) (Table 2 and Figure 2). Film microplastics collected were mostly transparent, with the exception of some that were colored (Figure 1B).

**TABLE 2:** Abundance of various microplastic types in different groups of shrimp.

**Species**	**Location**	**Number of fibers/g w.w.**	**Number of fragments/g w.w**	**Number of film/g w.w.**	**Number of spheres/g w.w.**
*L. vannamei*	Malaysia	1.20 ± 1.06	1.60 ± 1.72	205 ± 37.9	1.31 ± 0.275
	Ecuador	8.66 × 10^–3^ ± 6.77 × 10^–4^	0.861 ± 7.41 × 10^–2^	25.3 ± 1.59	8.93 × 10^–3^ ± 5.18 × 10^–4^
*P. muelleri*	Argentina, Southwest Atlantic, FAO 41	468 ± 104	4930 ± 1110	3190 ± 643	32800 ± 793
*F. indicus*	Indonesia, Eastern Indian ocean, FAO 57	1100 ± 51.5	4990 ± 334	8950 ± 515	21500 ± 805

The average weight of one individual of *P. muelleri* was 56 ± 4 g w.w. (Table 1). A total of 5,867,833 pieces of microplastics were obtained from 15 individuals of *P. muelleri*, with an average of 7050 ± 4178/g w.w. of microplastics. When examined, one shrimp individual had white colored spheres, which were visible from the outside of the intestine. These spheres were as large as 500 μm in diameter (Figure 1C). From this sample, spheres were the most abundant (69.6%), followed by fragments (21.5%), film (7.4%) and fibers (1.5%) (Table 2 and Figure 2). These spheres were mostly opaque and ranged from 10-20 μm in diameter (Figure 1F). An agglomeration of spheres was also observed in this sample (Figure 1D).

The average wet weight of one individual of *F. indicus* was 38 ± 1 g w.w (Table 1). In 18 individuals of *F. indicus*, 3,763,750 microplastic particles were recorded, with an average number of 5570 ± 100/g w.w. (Table 2). Microplastic spheres were the most abundant in this sample (61.6%), Film and fragment microplastics were 25.4% and 10.8% respectively. Fibers were the least abundant at 2.2% (Table 2; Figure 2). Blue colored fibers were observed in this sample (Figure 1E).

One-way ANOVA analyses demonstrated that the abundance of microplastic fibers, fragments, spheres film and spheres were significantly different when compared between species (*p*-value < 0.01; Table 3) and also within each shrimp type (p-value < 0.01; Table 4). Pairwise comparisons of the abundance of each microplastic type were made between species types via Tukey’s HSD *Post hoc* test (Table 5). The abundance of microplastic fibers, fragments, film and spheres were significantly different between shrimp pairs with a few exceptions. There was no significant difference between the abundance of the four microplastic types in *L. vannamei* from Malaysia and Ecuador (*p*-value > 0.01; Table 5). There was also no significant difference in the abundance of microplastic fragments collected from *P. muelleri* and *F. indicus* (*p*-value > 0.01; Table 5). The differences in the abundance of microplastic types within each shrimp were also examined (Table 6). In *L. vannamei* (Malaysia) and *L. vannamei* (Ecuador), significant differences in microplastic abundances were found between fibers vs film, fragments vs film and film vs spheres (*p*-value < 0.01; Table 6). There were significant differences between pairwise comparisons of all types of microplastics in both *P. muelleri* and *F. indicus* (*p*-value < 0.01; Table 6).

**TABLE 3:** Results from one-way ANOVA test for differences in the composition of ingested microplastics between the four shrimp types.

	**df**	**MS**	***F* value**	***P*-value**
Microplastic fiber	3	136 × 10^4^	886	<0.001*
Microplastic fragment	3	311 × 10^5^	1790	<0.001*
Microplastic film	3	868 × 10^5^	691	<0.001*
Microplastic sphere	3	134 × 10^7^	10500	<0.001*

**TABLE 4:** Results from one-way ANOVA test for differences in the composition of ingested microplastics within each shrimp type.

	**df**	**MS**	***F* value**	***P*-value**
*L. vannamei* (Malaysia)	3	51800	239	<0.001
*L. vannamei* (Ecuador)	3	785	2000	<0.001
*P. muelleri*	3	114 × 10^7^	10300	<0.001
*F. indicus*	3	389 × 10^6^	2120	<0.001

**TABLE 5:** Results of Tukey’s HSD *Post hoc* test in the composition of ingested microplastic particles between the four shrimp types.

	**Fiber**		**Fragment**		**Film**		**Sphere**	
	**Q statistic**	***P*-value**	**Q statistic**	***P*-value**	**Q statistic**	***P*-value**	**Q statistic**	***P*-value**
*L. vannamei* (Malaysia) vs. *L. vannamei* (Ecuador)	0.058	0.899	0.007	0.899	1.23	0.801	0.007	0.899
*L. vannamei* (Malaysia) vs *P. muelleri*	22.8	<0.001*	24.1	<0.001*	20.5	<0.001*	164	<0.001*
*L. vannamei* (Malaysia) vs. *F. indicus*	53.6	<0.001*	24.3	<0.001*	60.0	<0.001*	107	<0.001*
*L. vannamei* (Ecuador) vs. *P. muelleri*	22.8	<0.001*	24.1	<0.001*	21.7	<0.001*	164	<0.001*
*L. vannamei* (Ecuador) vs. *F. indicus*	53.6	<0.001*	24.4	<0.001*	61.2	<0.001*	107	<0.001*
*P. muelleri* vs. *F. indicus*	30.8	<0.001*	0.273	0.899	39.5	<0.001*	57.0	<0.001*

The *indicates significant differences between the species type compared (*p* < 0.01).

**TABLE 6:** Results of Tukey’s HSD *Post hoc* test in the composition of ingested microplastic particles within each shrimp type.

	***L. vannamei* (Malaysia)**		***L. vannamei* (Ecuador)**		***P. muelleri***		***F. indicus***	
	**Q statistic**	***P*-value**	**Q statistic**	***P*-value**	**Q statistic**	***P*-value**	**Q statistic**	***P*-value**
Fiber vs. fragment	0.128	0.899	2.38	0.365	16.7	<0.001*	21.7	<0.001*
Fiber vs. film	30.4	<0.001*	86.3	<0.001*	10.2	<0.001*	43.8	<0.001*
Fiber vs. sphere	0.016	0.899	9 × 10^–4^	0.899	121	<0.001*	114	<0.001*
Fragment vs. film	30.3	<0.001*	83.9	<0.001*	6.53	<0.001*	22.1	<0.001*
Fragment vs. sphere	0.112	0.899	2.38	0.365	104	<0.001*	91.9	<0.001*
Film vs. sphere	30.4	<0.001*	86.3	<0.001*	111	<0.001*	69.8	<0.001*

The *indicates significant differences between the microplastic type compared (*p* < 0.01).

## Discussion

This study demonstrated that microplastic particles are present in the digestive tracts of three species of shrimp, *L. vannamei, P. muelleri* and *F. indicus*, which are commercially available in the supermarkets of Singapore. Although microplastics are well-known to be a widespread contaminant in the marine environment, the presence of microplastics in commercially available shrimp has not yet been documented. This is the first study that has documented the presence of microplastics in these commercially available shrimps. The first report of microplastics in marine decapod shrimp species was the European brown shrimp, *Crangon crangon* of the Southern North Sea (Devriese et al., 2015). *C. crangon* had an average abundance of 1.23 ± 0.99 items/individual (Devriese et al., 2015; Table 7). Subsequently, the presence of microplastics has been investigated in five other marine decapod species including the Gamba shrimp (*Aristeus antennatus*), black tiger shrimp (*P. monodon)* and Indian white shrimp (*F. indicus)* (Table 7). These species have high commercial interest and are commonly consumed seafood by humans (Rosa and Nunes, 2004; Sriket et al., 2007). Although the same species *F. indicus* was compared from Kerala, India (Daniel et al., 2020) and FAO57, Indian Ocean of this study, both had different microplastic abundances and dominant ingested microplastic type (Table 7). This is likely due to the availability of microplastic type in the environment (Fossi et al., 2014). In the study of Daniel et al. (2020), microplastic fibers were dominant in *F. indicus* as those individuals were obtained from the coastal region with large amounts of fishing gear and fabric textiles (Kane and Clare, 2019; Robin et al., 2020). In the case of *F. indicus* from FAO57, spheres were the dominant microplastic type observed (Table 2). However, as FAO57 of the east Indian ocean extends from the Bay of Bengal to Southern Australia, the exact origin of *F. indicus* from this study is unknown and hence no further deductions can be made.

**TABLE 7:** Studies investigating microplastic presence in marine decapod species.

**Species**	**Location**	**Microplastic abundance**	**References**
European brown shrimp *(Crangon crangon)*	North Sea	1.23 ± 0.99 items/individual	Devriese et al., 2015
Gamba shrimp (*Aristeus antennatus)*	Balearic basin, northwestern Mediterranean sea	39.2% individuals reported to have ingested microplastics. Fibers dominant	Carreras-Colom et al., 2018
	Sardinia Island, Mediterranean Sea	1.66 ± 0.11 pieces/individual; Fragments dominant at 53%	Cau et al., 2019
Green tiger shrimp (*Penaeus semisulcatus)*	Northeast of Persian Gulf	0.360 pieces/g of muscle	Akhbarizadeh et al., 2019
Indian white shrimp *(Fenneropenaeus indicus)*	Cochin, Kerala, India	0.04 ± 0.07 pieces/individual Fibers dominant at 83%	Daniel et al., 2020
Black tiger shrimp (*Penaeus monodon)*	Northern Bay of Bengal, Bangladesh	6.60 ± 0.2 pieces/g of gastrointestinal tract Filaments dominant at 57%	Hossain et al., 2020
Brown shrimp (*Metapenaeus monoceros)*		3.87 ± 1.05 pieces/g of gastrointestinal tract Filaments dominant at 58%	
Whiteleg shrimp (*Litopenaeus vannamei)*	Malaysia	20.8 ± 3.57/g w.w. Film dominant at 97.9%	**This study**
	Ecuador	13.4 ± 1.42/g w.w. Film dominant at 93%	
Argentine red shrimp *(Pleoticus muelleri)*	Argentina Southwest Atlantic, FAO 41	7050 ± 4178/g w.w Spheres dominant at 69.6%	
Indian white shrimp *(Fenneropenaeus indicus)*	Indian ocean, FAO 57	5570 ± 100/g w.w. Spheres dominant at 61.6%	

Microplastic fibers, film, fragments and spheres were observed to be present in the GI tracts of shrimp species. Among the species, *L. vannamei* consumed the least number of microplastics per wet weight regardless of location. Furthermore, there were no significant differences among the different microplastic types in *L. vannamei* from Malaysia and Ecuador. *P. muelleri* and *F. indicus* consumed approximately 200 times more microplastics compared to *L. vannamei* per wet weight of individual. Both groups of *L. vannamei* from Malaysia and Ecuador consumed similar concentrations of microplastics. As shrimps are a detritus feeder (Varadharajan and Pushparajan, 2013), this could mean that the abundance of microplastics in the benthic sediments of Malaysia and Ecuador could be similar. The number of ingested microplastics can reflect the abundance of microplastics present in the environment organisms exist in (Fossi et al., 2014; Nel et al., 2018). The abundance of microplastics have been measured in the surface marine waters of Malaysia, at 0.13–0.69 particles/L (Khalik et al., 2018). However, the abundance of microplastics in benthic marine sediments has not yet been studied in Malaysian waters. Currently, no records exist regarding microplastics in the marine waters of Ecuador. In addition, the similar concentrations of microplastics recorded in *L. vannamei* shrimp from Malaysia and Ecuador could also be due to similar filtration and ingestion rates in both groups of shrimp. In this study, *P. muelleri* was sourced from the Southwestern Atlantic and contained significantly more microplastics more than *L. vannamei.* The surface water samples of the Southwestern Atlantic had a concentration of 42,600–113,600 particles/L (Severini et al., 2019), which is about 160,000-300,000 times the concentration of that in Malaysian waters. Microplastic fibers were the most abundant microplastic recorded from the surface waters of the Southwestern Atlantic (Severini et al., 2019). However, the dominance of microfibers was attributed to the high level of harbor activities, which involved boating and fishing. Given that the composition of microplastics differs based on location (La Daana et al., 2017). The greater abundance of microplastics in the Atlantic Ocean could be reflected in the increased uptake and hence microplastic abundance in *P. muelleri* shrimp.

In *P. muelleri* and *F. indicus* shrimp, spheres were the most abundant type of microplastics. These spheres are likely to be microbeads, which are primary microplastics that are found in cosmetic or personal care products. Due to the small size of these particles, a significant proportion of these microbeads are discharged in the final effluent even after sewage treatment. In addition, these microbeads are non-biodegradable and hence exist in the marine environment for hundreds of years (Olesen et al., 2017). As a result, fouling can take place on the surface of these plastic particles, resulting in them sinking and being deposited on the seafloor (Kaiser et al., 2017). This results in the seafloor being a major sink for microplastics (Fang et al., 2018). Hence, it is common that marine organisms, especially bottom-dwelling detritus feeders, ingest these microbeads. The agglomeration of microbeads was also observed in *P. muelleri* shrimp (Figure 1D). Due to the interaction of salt with natural organic matter in seawater, it is not uncommon for microbeads in marine environments to aggregate together (Gambardella et al., 2017). Although a very large number of microplastics were observed in *P. muelleri* and *F. indicus* individuals, this was primarily due to the predomination of spheres, which were approximately 15–20 μm in diameter.

Although various types of frozen seafood such as clams, mussels, squids and fish are available in the supermarkets of Singapore, only three species of shrimp have been examined for their presence of microplastics to date. In other studies, mussels (*Mytilus edulis*) were examined from the supermarkets in the United Kingdom at 0.7 items/g (Li et al., 2018), mussels (*Mytilus edulis*; 0.36 items/g) and oysters (*Crassostrea gigas*; 0.47 items/g) from coastal farms in Europe (Van Cauwenberghe and Janssen, 2014). Scallops *(Argopecten purpuratus)* obtained from markets in Lima, Peru showed a mean abundance of 2.25 items/individual (De-la-Torre et al., 2019). Market oysters (*C. gigas*), mussels (*M. edulis*), clams (*Tapes philippinarum)* and scallops (*Patinopecten yessoensis*) from South Korea showed a mean microplastic concentration of 0.15 items/g (Cho et al., 2019). Analysis of bivalves from a market in Shanghai, China showed an abundance of up to 10.5 items/g (Li et al., 2015). For data comparison purposes, it is recommended that the method of microplastic analysis be taken into consideration, such as the chemical treatment for tissue digestion, density separation technique and the mode of microplastic identification (Cho et al., 2019). This is because the variation in the techniques of microplastic analysis can influence the abundance of microplastics obtained. Although the methods of analyses were not similar, these studies provide evidence that species of seafood from different parts of the world are contaminated by microplastics in the ocean. This highlights the pervasiveness of marine microplastic pollution and suggests that there are greater implications on food security and human health.

The presence of microplastics in seafood has led to increased concerns regarding its impact on human health (Sharma and Chatterjee, 2017; Smith et al., 2018). Before human consumption, shrimp are usually peeled to remove head and shell. However, the GI tracts of these organisms are not always completely removed, and hence the microplastics that are present in the intestines of shrimp could be passed on to humans through consumption. This is a route of exposure of microplastics to humans that is often discussed in relation to human health and food security (Barboza et al., 2018; Smith et al., 2018; Cox et al., 2019). Microplastics have shown to leach harmful additives (Koelmans et al., 2014; Hermabessiere et al., 2017) and accumulate persistent organic pollutants (POPs) such as phthalates and Bisphenol A (Rochman et al., 2013). Laboratory studies conducted on lugworms have shown that plastic additives are transferred from microplastics to the organism, resulting in a behavioral change (Browne et al., 2013). In another study, Lassen et al. (2015) showed that ingested microplastic particles from toothpaste can be absorbed by the human gastrointestinal tract. However, there are not yet any published studies examining the fate of microplastics from ingested seafood in humans. Hence, more information is required to ascertain the retention and the impact of microplastics from seafood on the human body.

## Conclusion

In this study, microplastics were identified in three species of shrimp, *L. vannamei, P. muelleri* and *F. indicus*, from four locations, that were obtained from the local supermarket of Singapore. Microplastic fibers, film, fragments and spheres were found in the GI tracts of shrimps. *F. indicus* has the greatest microplastic abundance per wet weight, followed by *P. muelleri* and *L. vannamei* shrimps. This could be reflected by the abundance of microplastics of benthic sediments where they were obtained. The results of this study provide novel evidence that microplastics exist in shrimps. The ingestion of these shrimp is a route of human exposure to microplastics as these organisms are often eaten whole without gut removal. Additional comprehensive surveys of supermarket seafood are crucial to assess the additional routes of microplastic exposure in humans. The findings of this study highlight the pervasiveness of microplastic pollution in commercially available seafood and provide crucial information for fisheries and the aquaculture industry.

## Data Availability Statement

The datasets presented in this study can be found in online repositories. The names of the repository/repositories and accession number(s) can be found in the article/supplementary material.

## Author Contributions

All authors listed have made a substantial, direct and intellectual contribution to the work, and approved it for publication.

## Conflict of Interest

The authors declare that the research was conducted in the absence of any commercial or financial relationships that could be construed as a potential conflict of interest.
